# Lymphoma: Immune Evasion Strategies

**DOI:** 10.3390/cancers7020736

**Published:** 2015-04-30

**Authors:** Ranjan Upadhyay, Linda Hammerich, Paul Peng, Brian Brown, Miriam Merad, Joshua D. Brody

**Affiliations:** 1 Division of Hematology and Medical Oncology, Icahn School of Medicine at Mount Sinai, New York, NY 10029, USA; E-Mails: ranjan.upadhyay@mssm.edu (R.U.); linda.hammerich@mssm.edu (L.H.); paul.peng@mssm.edu (P.P.); 2 Department of Genetics and Genomic Sciences, Icahn School of Medicine at Mount Sinai, New York, NY 10029, USA; E-Mail: brian.brown@mssm.edu; 3 Department of Oncological Sciences, Icahn School of Medicine at Mount Sinai, New York, NY 10029, USA; E-Mail: miriam.merad@mssm.edu

**Keywords:** checkpoint blockade, cytokines, immune escape, immunosuppression, immunotherapy, lymphoma, MDSC, Treg, TAM

## Abstract

While the cellular origin of lymphoma is often characterized by chromosomal translocations and other genetic aberrations, its growth and development into a malignant neoplasm is highly dependent upon its ability to escape natural host defenses. Neoplastic cells interact with a variety of non-malignant cells in the tumor milieu to create an immunosuppressive microenvironment. The resulting functional impairment and dysregulation of tumor-associated immune cells not only allows for passive growth of the malignancy but may even provide active growth signals upon which the tumor subsequently becomes dependent. In the past decade, the success of immune checkpoint blockade and adoptive cell transfer for relapsed or refractory lymphomas has validated immunotherapy as a possible treatment cornerstone. Here, we review the mechanisms by which lymphomas have been found to evade and even reprogram the immune system, including alterations in surface molecules, recruitment of immunosuppressive subpopulations, and secretion of anti-inflammatory factors. A fundamental understanding of the immune evasion strategies utilized by lymphomas may lead to better prognostic markers and guide the development of targeted interventions that are both safer and more effective than current standards of care.

## 1. Introduction

Advances in the past decade have compelled cancer biologists to modify the underlying classical principles previously used to define the pathogenesis of cancer, such as sustained proliferative signaling and insensitivity to growth suppressors [1]. One such emerging hallmark of cancer is its ability to evade destruction by the immune system [2]. There is significant evidence showing that both innate and adaptive immunity play crucial roles in eliminating nascent transformed cells [3]. However, these same protective mechanisms apply a selective pressure to shape the inherent immunogenicity of the developing tumor. These paradoxical anti- and pro-tumorigenic roles of the immune system form the basis for the immunoediting hypothesis [4,5,6], which proposes a model for how a malignancy develops the ability to escape the immune response.

Lymphomas, often originating from genetic alterations that bring an oncogene under the control of an Ig locus during physiological remodeling of the immunoglobulin genes, are no different [7]. If these precancerous cells overcome intrinsic tumor suppression mechanisms, they are frequently recognized and eliminated by the immune system, a process called “immune surveillance” [8]. Evidence for this process can be seen in immunodeficient mouse models, such as those lacking T cell and NK cell effector pathways. For example, mice lacking perforin or IFNγ developed spontaneous lymphomas that were immediately rejected by CD8+ T cells when transplanted into WT mice [9,10]. Therefore, development of lymphomas in an immunocompetent host requires genetic alterations that allow it to evade the immune system, either by avoidance of detection or by active disablement and elimination of immune effector cells. In this review, we discuss these immune evasion strategies, including molecules expressed at the surface of the cell, soluble factors that are secreted by the tumor, and cellular constituents that are recruited to maintain an immunosuppressive microenvironment.

## 2. Surface Molecules

### 2.1. Antigen Presentation Machinery

A common strategy for tumors to escape recognition by effector T cells is through generation of new tumor antigen variants, a result of their inherent genetic instability [11,12]. However, these neo-antigens remain potentially immunogenic targets for distinguishing them from normal cell counterparts. Hence, one of the most common genetic alterations involved in avoiding immune recognition is the downregulation or complete loss of the antigen presentation machinery. Immunohistochemical analysis indicates that more than half of extranodal diffuse large B cell lymphoma (DLBCL) samples show complete or partial loss of HLA class I expression, with similar results for class II expression [13]. These abnormalities can occur both through defects in the MHC loci or in the downstream processing machinery. For example, 75% of DLBCL samples, including both patient biopsies and cell lines, were found to lack membrane HLA class I expression due to aberrant expression patterns of β2-microglobulin [14]. Exome sequencing revealed disruption of both B2M alleles in 13 out of 111 DLBCL biopsies [15], and similar analysis determined B2M to be the most frequently mutated gene in classical Hodgkin’s lymphoma (cHL) [16]. Interestingly, 61% of biopsies concurrently lacked surface expression of CD58, a known ligand of the CD2 protein involved in the activation of natural killer (NK) cells as well as cytotoxic T lymphocytes (CTLs), suggesting active co-selection for clones that will evade both adaptive and innate immune surveillance [14]. Similarly, the non-classical molecule HLA-G, often associated with immune tolerance during pregnancy and protection from NK cell-mediated cytotoxicity, has also been shown to be expressed in 54% of cHL cases in conjunction with loss of MHC class I [17]. Proteins involved in antigen processing such as LMP2/7 [18] or antigen transport such as TAP1 [13] can also be downregulated, leading to ineffective presentation to infiltrating CD8+ T cells.

B cell malignancies are exceptional amongst cancers in that they are derived from antigen-presenting cells that express MHC class II and can potentially present tumor-associated antigens to CD4+ T cells. Therefore, downregulation of MHC class II genes is also a major mechanism of immune evasion in lymphoma. IHC of cHL biopsies showed that lack of HLA class II expression coincided with extranodal disease and adverse clinical outcome [19]. Indeed, similar observations were found for DLBCL biopsies; decreased class II expression correlated with fewer tumor-infiltrating lymphocytes (TILs) and poorer 5-year overall survival [20,21]. In addition to homozygous deletion [13], two other mechanisms of MHC-II downregulation have been identified. One is the rearrangement of the CIITA gene (a transactivator of MHC-II), found in 38% of primary mediastinal B cell lymphomas (PMBCL) and in 15% of cHL, leading to not only the subsequent downregulation of class II but also the overexpression of PD-L1 and PD-L2 due to fusion of the CIITA promoter region [22]. Interestingly, mutations in *CREBBP*, a chromatin-modifying gene with a well-characterized role in CIITA-dependent expression of MHC class II, were highly enriched in progenitors of follicular lymphoma (FL), indicating the importance of immune evasion in early tumorigenesis [23]. An additional mechanism is the partial differentiation of post-germinal activated B cell DLBCL towards a more plasmablastic lymphoma (PBL) phenotype, resulting in reduced class II expression [24].

### 2.2. Costimulatory and Checkpoint Molecules

Once a tumor cell has been recognized by its cognate lymphocyte, manipulation and dysfunction of the costimulatory pathways is another major mechanism of failed anti-tumor immunity. The B7 family of molecules is one of the most important mediators of this second signal after antigen recognition. Physiologically, this signal exists to maintain a balance between stimulating a potent response and suppressing potentially detrimental autoimmunity [25]. Expression levels of B7-1 (CD80) and B7-2 (CD86), ligands for the activation receptor CD28 and inhibitory receptor CTLA-4 (CD152) on T cells, vary between different models and subtypes of lymphoma. In the murine A20 lymphoma model, cells express no detectable surface CD80 [26]. Additionally, loss of CD86 expression in DLBCL samples is associated with decreased tumor-infiltrating lymphocytes and subsequently shorter relapse-free survival [27]. In contrast, malignant Hodgkin Reed-Sternberg (HRS) cells were shown to universally express high levels of CD80/86 [28]. Of note, however, is that CTLA-4, which is upregulated on activated T cells, can bind CD80/86 with higher affinity and avidity than CD28 [25]. Therefore, while these molecules are clearly involved in controlling immune activation, their roles in the progression of lymphoma may depend not only on the specific ligand [29] and receptor but also on contextual cues from the microenvironment.

Another receptor that acts as a bidirectional molecular switch is herpesvirus entry mediator (HVEM, TNFRSF14), a molecule that interacts with several ligands, including B- and T-lymphocyte attenuator (BTLA, CD272), TNFSF14 (CD258, LIGHT), and CD160 on NK and T lymphocytes [30]. HVEM is of particular interest because it was found to be recurrently mutated in DLBCL via whole-exome sequencing [31]. Additionally, 18% of FL patients had nonsynonymous mutations affecting HVEM, correlating with inferior clinical outcomes and suggesting a tumor suppressor role for the molecule [32]. This was corroborated *in vitro* when incubation of mantle cell lymphoma (MCL) cells with LIGHT-transfected cells resulted in increased expression of Fas and susceptibility to Fas-induced apoptosis [33]. However, HVEM can also clearly send an inhibitory signal and promote immune tolerance when bound to BTLA and CD160 [30,34], suggesting that the aforementioned mutations may only affect LIGHT-binding or LIGHT-mediated effector sites on HVEM. While the BTLA-HVEM pathway as a mechanism of immune escape is only beginning to be studied in the context of lymphoma [35], it may be an actionable target similar to the CTLA-4 inhibitory pathway.

The roles of B7-H1 (CD274, PD-L1) and B7-DC (CD273, PD-L2) in lymphoma are far less ambiguous. Upon binding to PD-1 (CD279) on activated T cells, the effect is profoundly inhibitory, including promotion of apoptosis and anergy as well as induction of immunosuppressive cytokines [36]. Several groups have shown this pathway to be a prominent mechanism of immune resistance in lymphoma patients. High PD-L1 and PD-L2 expression was demonstrated in primary HRS cells via IHC, with congruent expression of PD-1 in infiltrating T cells [37]. Interestingly, these patients also had significantly elevated PD-1 expression in peripheral T cells during active disease compared to those of healthy controls, suggesting a systemic effect that declined with treatment [37]. Gene expression studies on PMBCL and cHL patient samples also revealed select amplification of the genetic loci-encoding PD-1 ligands [22,38] and JAK2, which can further induce transcription of these ligands [39]. PD-L1 expression was similarly found in various subsets of B and T cell lymphomas [40,41], and the blocking of PD-L1 was found to enhance proliferation and inflammatory cytokine secretion by autologous T cells [42].

### 2.3. Effector Molecules

Once activated CTLs engage their cognate tumor cells, one of the main mechanisms by which they induce apoptosis is via the FasL-FasR (CD95L-CD95) interaction. In an immunodeficient mouse model, only transfer of CD8+ T cells deficient in FasL impaired the elimination of B cell lymphomas, while transfer of CD8+ T cells with deficiencies in perforin, granzymes, TRAIL, or IFNγ had no effect [43]. Additionally, B cell lymphomas that developed in T cell-sufficient mice expressed lower levels of FasR compared to their counterparts in T cell-deficient mice [43]. These observations indicate that the FasL-FasR interaction is important in CTL-mediated killing of lymphomas, and these tumors can gain resistance to apoptosis by downregulation of FasR. This hypothesis is supported by clinical evidence that lower levels of FasR in germinal-center-type DLBCL is associated with significantly lower overall survival, with the same trend observed for overall DLBCL cases [44]. HL-derived cell lines and primary HRS cells were also found to have high expression of cellular FLICE-inhibitory protein (c-FLIP), which protects against Fas-mediated death and may be another method of immune evasion in this pathway [45,46,47].

Interestingly, T cells also upregulate FasR upon antigenic activation and expansion. Tumors can potentially hijack this regulatory mechanism by upregulating FasL expression and inducing apoptosis of infiltrating lymphocytes. This was demonstrated for the first time by co-culture of a FasL+ T cell lymphoma line with its cognate FasR+ CTL clone [48]. The resulting apoptosis in both cell types validated that the FasL-FasR interaction can be bidirectional, and the overall effect may depend on respective expression levels or other extrinsic factors. Indeed, IHC and western blotting of HL tumor tissue showed high FasL expression in HRS cells, indicating a potential immune escape mechanism [49].

In addition to disruption of cytolysis, an emerging story in lymphomas is the ability to upregulate CD47, a marker of self, and inhibit phagocytosis of the tumor cell via the CD47-SIRPα pathway in host phagocytes [50]. Blockade of this signal via anti-CD47 antibody in primary human xenotransplant mouse models of DLBCL and FL showed both significant reduction of tumor burden and increased survival [51]. Subsequent studies on the same model indicated that CD47 expression is increased in disseminated disease, and blockade of the CD47-SIRPα pathway can prevent extranodal involvement [52]. Overexpression of CD47, therefore, seems to protect tumor cells from innate immune attack, in addition to potential downstream adaptive mechanisms that may be involved in the priming of a T cell response [53]. A different type of immune effector molecule appropriation by tumor cells is observed with Toll-like receptors (TLR). During a normal innate immune response, TLR triggering results in the production of inflammatory cytokines; however, other TLR downstream pathways involving MAPK, NF-κB, and PI3K can also potentially contribute to cell survival and tumor progression. In the case of MCL, it has been observed that stimulation of TLR4—Which was found to be highly expressed in primary MCL samples and cell lines—Resulted in tumor cell proliferation as well as upregulated secretion of soluble immunosuppressive factors IL-6, IL-10 and VEGF [54].

An additional surface-mediated mechanism of immune evasion is the demonstration of impaired immunological synapses induced by contact with FL cells [55]. Inhibition of F-actin formation and polarization was found only in TILs, and this effect was reproduced *in vitro* by co-culture of previously healthy T cells with FL cells [55]. Similar changes in the cytoskeletal machinery leading to dysfunctional synapse formation were observed in chronic lymphocytic leukemia-associated T cells [56,57,58]. While the exact mechanism is unclear, an siRNA screen by the same group revealed that CD200, B7-H3, and the aforementioned PD-L1 and HVEM were all involved in this impairment of T cell actin dynamics [59].

### 2.4. Therapeutic Potential

The most successful example to date of translating immune evasion strategies into anti-cancer therapies is the blockade of T cell checkpoint molecules like CTLA-4 or PD-1. Treatment with the anti-PD1 antibody pidilizumab in DLBCL patients who had measurable disease after autologous hematopoietic stem cell transplant resulted in an overall response rate of 51% [60]. Similarly, 66% of relapsed FL patients responded favorably to a combination of pidilizumab and rituximab, with an impressive 52% of patients having a complete response, exceeding the expectation with rituximab monotherapy [61]. PD-1 blockade with nivolumab demonstrated significant therapeutic activity for relapsed or refractory cHL with an overall response rate of 87% [62,63], and preliminary results indicate 30 to 40% response rates for DLBCL and FL as well [64]. Preliminary reports on pembrolizumab also demonstrate greater than 50% overall response rates for patients with relapsed or refractory cHL [65].

Unfortunately, the responses to anti-CTLA-4 have been more modest [66,67,68], but recent data indicates that higher doses may be well-tolerated and provide more clinical benefit [69]. Interestingly, studies in melanoma suggest that a potential combination therapy against both CTLA-4 and PD-1 could surpass the objective response rates of either alone [70].

## 3. Cellular Regulation

### 3.1. Regulatory T Cells

The tumor microenvironment of established lymphomas is a complex mixture of neoplastic and non-malignant cells. The latter population is rich in not only stromal cells of mesenchymal origin that provide structural support and pro-tumorigenic signaling but also tolerogenic immune cells that can potentially suppress the host’s response to the tumor [71]. In fact, the gene signatures of these tumor-infiltrating immune cells were found to have strong predictive value for clinical outcome in patients with follicular lymphoma [72]. Perhaps the most well-studied of these populations in lymphoma is the regulatory T cell (Treg), a physiologically suppressive subset that serves to limit autoimmune responses but is often hijacked by tumors to promote tolerance. The proportion of CD4+CD25+FoxP3+ Tregs in the periphery seems to increase with tumor burden, and depletion of this population prior to challenge in the A20 lymphoma mouse model resulted in 70% tumor-free survival [26]. Interestingly, this effect was not seen in depletion studies following the establishment of a palpable tumor, suggesting an integral role in early development of lymphomas [26]. Systemic induction of Tregs was also observed clinically, as CD4+CD25+FoxP3+CD127 low cells were markedly increased in the peripheral blood of NHL patients and correlated with tumor burden [73]. Unsurprisingly, Tregs were found to be overrepresented in both NHL [74] and HL [75] biopsy specimens as well.

Numerous *ex vivo* studies have confirmed the immunosuppressive role of Treg in the lymphoma microenvironment. HL-infiltrating lymphocytes, predominantly IL-10-secreting and CD4+CD25+ Tregs, were found to be unresponsive to stimulation with cognate antigens or even nonspecific mitogens, and this anergic phenotype was extended to autologous PBMCs after co-culture with these cells [75]. Intratumoral CD4+CD25+ Tregs in NHL specimens not only directly inhibited proliferation of autologous infiltrating CD8+ T cells, but also prevented both production and degranulation of perforin and granzyme B, molecules critical to their anti-tumor cytolytic functions [76]. Similar functional deficiencies were observed after co-culture with infiltrating CD4+ T cells, including inhibited proliferation and dampened IFNγ and IL-4 production, cytokines that mediate anti-tumor activity [74]. In mice, transfer of these Treg were even potent enough to eliminate any protective effects seen in a previously successful adoptive transfer immunotherapy model [26]. Clinically, the capacity of NHL cells to induce Treg was inversely correlated with clinical outcome after receiving an *in situ* vaccine [77].

Precisely how these Treg are recruited to—Or induced in—The tumor remains unclear. *Ex vivo* studies of NHL patient samples revealed that direct cell contact with tumor cells induced differentiation of autologous PBMCs into CD4+CD25+FoxP3+ Tregs that were found to be directly responsible for effector T cell hyporesponsiveness by *in vitro* depletion experiments [73,78]. The interaction between costimulatory molecules and their ligands seems to play a role in this immunosuppressive skewing. Blockade of the CD70 or CD80/86 pathways during co-culture with lymphoma cells reversed the differentiation of CD4+ cells into Tregs, and instead promoted the generation of anti-tumor Th17 cells [79]. Similarly, blockade of the PD-L1/PD-1 interaction seems to restore some proliferative capacity for infiltrating T cells [74]. Cytokines and chemokines, discussed in more detail below, also play a crucial role in the chemotaxis and differentiation of Tregs.

### 3.2. Tumor-Associated Macrophages

Myeloid-derived regulatory cells comprise the second major population of non-malignant cells that contribute to the development of lymphomas. Of these, the tumor-associated macrophage (TAM) is one of the most abundant cell types in the microenvironment, and in the alternatively activated (M2-polarized) state can serve as a key component of the inflammatory circuits that promote tumor cell invasion, angiogenesis, and immunoregulation [80]. In reality, the tumor probably contains a mosaic of macrophages in a complex spectrum of activated states, but the overall effect seems to be immunosuppressive [81]. Evidence certainly suggests that lymphoma-associated macrophages are no different. High CD68+ macrophage content in FL biopsy specimens was shown to be a significant negative predictor of overall and progression-free survival [82]. Similarly, an increased number of CD68+ macrophages in cHL biopsies correlated strongly with a shortened survival and could even be used as a clinical predictor for relapse after therapy, validating the finding that biopsies from patients that failed to respond to primary treatment overexpressed a macrophage gene signature [83]. Histological staining for M2-polarized macrophages (CD68+CD163+) was an even better negative predictor of overall survival for DLBCL patients than total CD68+ macrophage content, while M1-polarized cells had no predictive value at all [84]. Depletion of M2-macrophages in a preclinical model of cutaneous T cell lymphoma (CTCL) showed markedly less tumor growth [85]. While there is little published data on the mechanisms of macrophage recruitment and TAM-mediated immunosuppression in the context of lymphoma, what we have learned about them from other models has been recently reviewed by Noy *et al.* [81].

### 3.3. Myeloid-Derived Suppressor Cells

Myeloid-derived suppressor cells (MDSC), classically defined in mice as CD11b+Gr1+ cells, comprise another key pathological population driven by tumor-derived factors that has demonstrated potent ability to blunt anti-tumor CTL responses by nutrient depletion, generation of oxidative stress, interference of lymphocyte trafficking, and activation of Tregs [86]. The role of MDSCs in lymphoma is only beginning to be explored, but preclinical evidence has emerged suggesting this population to be a major driver of tolerance. In the A20 lymphoma mouse model, MDSCs induced activation and proliferation of antigen-specific Tregs, leading to suppression and anergy of anti-tumor effector T cells [87]. Furthermore, this group demonstrated that the majority of tumor antigen was uptaken by cells with an MDSC surface phenotype, suggesting that these cells not only actively induce tolerance but also passively limit the amount of antigen that can be processed by other professional antigen-presenting cells like dendritic cells (DCs) [87]. Consistent with these findings, depletion of MDSCs in a mouse model of lymphoma inhibited tumor growth [88].

The accumulation of MDSCs, similar to Tregs and macrophages, is usually due to tumor-derived factors. Another source of these cytokines, including TGFβ and IL-13, seems to be type II (*i.e.*, non-classical) NKT cells, at least in a mouse model of B cell lymphoma [89]. Increased tumor incidence in mice deficient of type I (*i.e.*, invariant/Vα14-restricted) NKT cells, in addition to decreased tumor incidence in mice deficient in both types of NKT cells, suggests an innate immunosuppressive role for type II NKT cells intimately tied to MDSCs [89].

Amongst myeloid-derived cells, DCs are crucial initiators of the adaptive immune response and have been shown to correlate with survival in patients with lymphoma [90]. Preclinical models have consequently demonstrated that the DC growth factor Flt3L induces tumor protection [91,92]. Their functional impairment (e.g., reduced expression of costimulatory molecules, production of Th2 cytokines, and deficient chemotaxis) has also been shown in multiple solid cancers as a method of immune evasion [86]. While there have been few direct studies on DC phenotypes and their ability to prime T cells in the context of lymphomas, data demonstrating a decrease in expression of lymph node homing molecules on tumor-associated DCs in NHL biopsies suggests that mechanisms analogous to those found in other cancers are possible [93].

In addition to local effects by cellular infiltrates, one study has also implicated lymphoma in systemic immunosuppression driven by myeloid-derived cells. These CD14+HLA−DRlow/-monocytes were found in increased ratios in the peripheral blood of NHL patients compared to healthy controls, and levels correlated with aggressiveness of the disease [94]. Impaired proliferation and IFNγ production was reversed by removal of these cells, which sometimes reached as high as 70% of all circulating monocytes [94]. The mechanism of immunosuppression seems to be primarily through arginine metabolism, but the origins of this population remain unclear, including whether a relationship exists between these cells and lymphoma-associated macrophages within the tumor microenvironment.

### 3.4. Stromal Cells

Immune tolerance to lymphomas does not necessarily have to be mediated by immune cells. For example, T cell immunoglobulin and mucin domain-containing molecule 3 (Tim-3), which can directly induce tolerance in CD4+ T cells and inhibit Th1 polarization needed for an anti-tumor response, was found to be preferentially expressed on lymphoma-derived endothelial cells [95]. Therefore, lymphoma neovasculature has the potential to act not only as a vessel system but also as a functional immunological barrier that effectively hides the tumor from effector cells. Unsurprisingly, expression of Tim-3 directly correlated with disseminated disease and poor prognosis [95].

### 3.5. Therapeutic Potential

Lymphoma therapies targeting the activity of these immunosuppressive cells have shown promise. Denileukin diftitox is a fusion protein consisting of human IL-2 and the cytotoxic domain of diphtheria toxin, allowing for directed lethal activity against CD25+ cells, including both malignant cells and Tregs. Already approved by the FDA for CD25+ cutaneous T cell lymphomas [96], denileukin diftitox has an overall response rate of 25% as a single agent in relapsed or refractory B cell NHL [97], and its combination with rituximab saw an increase to 32% [98]. However, depletion of CD25+ Tregs in advanced-stage FL patients provided no added benefit over treatment with rituximab alone, and an increase in serum IL-10 and CXCL10 after combination therapy actually resulted in more adverse events [99]. Another study demonstrated clinical responses in only 10% of low-grade NHL patients, indicating very modest efficacy in this patient population [100].

The toxicities of these agents call into question the merits of such drastic strategies, like depleting an entire population of cells. More recent successful immunotherapies have attempted to slightly modify the immune response at different stages in order to produce a synergistic effect. Lenalidomide is one such pleiotropic drug that has shown promise against lymphomas in clinical trials and is FDA approved for MCL. In addition to its potent anti-angiogenic effects and direct cytotoxic effects on malignant cells, it has also been classified as an immunomodulatory drug due to its abilities to enhance innate immunity, polarize towards a Th1 response, reduce systemic levels of suppressor cells, and even repair T cell immunologic synapse dysfunction [55,59,101]. In an international phase II trial, lenalidomide as a single agent had an overall response rate of 35% against relapsed or refractory B cell NHL [102]. Combination with rituximab appears to be synergistic and highly effective; 90% of previously untreated advanced-stage low-grade nHL patients achieved a clinical response [103]. Preclinical models suggest that this effect may be due to lenalidomide-mediated activation of DCs and increased recruitment of NK cells to carry out antibody-dependent cellular cytotoxicity (ADCC) at the tumor site [104,105]. Multiple phase I/II trials are underway for combining lenalidomide with the standard of care R-CHOP in the hopes that this may improve long-term outcomes [106,107,108,109].

## 4. Soluble Factors

### 4.1. IDO, Galectins, and Prostaglandins

Extracellular molecules play a crucial role in shaping the immune milieu of the tumor. One of the key mechanisms by which the aforementioned TAMs, MDSCs, and immature DCs regulate T and NK cell activity is by metabolism of extracellular tryptophan, leading to both depletion of an essential nutrient and accumulation of immunosuppressive degradation products. High expression of the pathway’s rate-limiting enzyme indoleamine 2,3-dioxygenase (IDO) has been found in tumor cells of both mouse models of lymphoma [26] and DLBCL samples with poor response to chemotherapy [110]. In addition to direct apoptotic effects on effector T cells, mouse models demonstrate that intrasplenic injection of lymphoma cells also induces expansion of CD4+CD25+ Tregs that is reversible with an IDO inhibitor [111]. Inducible expression of IDO in mesenchymal stem cells also dramatically promotes lymphoma growth while reducing infiltrating lymphocytes [112], consistent with the role that mesenchymal stem cells have been shown to play in lymphoma pathogenesis [113,114]. Thus, IDO expression can be found in a variety of cell types, and its effect on tryptophan within the microenvironment seems to be a major mechanism of immune subversion.

Other secreted immunomodulatory molecules include the glycan-binding protein galectin-1 (gal-1) and the lipid prostaglandin E2 (PGE2). Gal-1 was found to be overexpressed by HRS cells within cHL tumors, and RNA interference studies revealed that gal-1 was directly responsible for the Th2 polarization and the expansion of Tregs within the tumor microenvironment [115]. Additionally, high expression of gal-1 in cHL patients is associated with reduced infiltration of CD8+ T cells, and *in vitro* stimulation with gal-1 results in decreased T cell proliferation and IFNγ production [116]. PGE2 is an interesting molecule in that it has a direct suppressive effect on B cell proliferation through the EP4 receptor [117] and can also inhibit the activation of T cells by disruption of the TCR signaling pathway [118]. Transcriptional analysis confirmed that the CD4+ T cells found in cHL are under the influence of PGE2, and these effects account for at least part of the impaired immune functions associated with the tumor [118].

### 4.2. Decoy Proteins

Soluble decoy proteins may be another important strategy for evading anti-tumor immunity. Decoy receptor 3 (DcR3), which can bind to FasL and prevent it from mediating apoptosis by cytotoxic T cells, was found to be amplified in virus-associated lymphomas [119]. In a subsequent study, DcR3 expression was found to be a key mechanism of chemotherapy resistance in DLBCL and correlated with poor clinical outcome [120]. The serum concentration of soluble Fas ligand, which has a similar mechanism as a decoy for the Fas-mediated pathway, was also significantly elevated in patients with aggressive NHL [121]. There is also evidence that lymphomas can develop resistance to NK cell-mediated immunity by secreting NKG2D ligand, a decoy for an activating receptor on NK cells [122]. In addition to directly inhibiting induction of apoptosis, decoy proteins can also serve as molecular sinks for cytokines involved in proper T cell activation. An example of this strategy is the expression of soluble IL-2Rα, which can effectively reduce the IL-2 available for the development of effector T cells. Serum sIL-2Rα levels in cHL patients at diagnosis correlated with not only tumor burden but also poor response to treatment [123]. This is not surprising given that high sIL-2Rα levels inhibited mitogen-induced proliferation in normal lymphocytes, and this effect was reversed with supplementation of IL-2 [124]. Unfortunately, the idea that cytokines have a singular effect and can be easily targeted for therapy is too simplistic. Cytokine signaling is highly dependent upon microenvironmental context, as demonstrated by the fact that the blockade of IL-2 in another study decreased the generation of Tregs and reversed the immunosuppressive skewing of lymphocytes from NHL specimens [79].

### 4.3. Cytokines

Cytokines are a crucial component of immune regulation, and there is evidence of the role of many cytokines in the pathology of lymphomas as well. IL-10 and TGFβ, in particular, have potent immunosuppressive properties. The sources of many of these cytokines are the aforementioned cellular populations that are recruited to the lymphoma microenvironment. For example, a large proportion of infiltrating lymphocytes in HL specimens were determined to be induced Tregs that express IL-10 [75]. MDSCs are also known to secrete numerous immunosuppressive cytokines, and M2-polarized tumor-associated macrophages are even defined by their ability to express IL-10, so the abundance of these cytokines in the tumor microenvironment is unsurprising [86]. Of greater interest is the ability of these malignant B cells to secrete these cytokines themselves, since this indicates a novel and more direct strategy for evading the immune system. Indeed, nearly half of all tested NHL samples stained positive for IL-10 expression, and all patients that had detectable levels of IL-10 in *ex vivo* cultures also had elevated serum levels of IL-10, indicating that the tumor cells had been the major source of the cytokine [125]. In fact, phenotypic analysis of CLL cells demonstrated many functional similarities with the IL-10-producing regulatory B (B10) cell subset [126]. These same B10 cells were previously shown to inhibit antibody-mediated immune recognition and clearance of lymphoma [127].

Similarly, TGFβ is also expressed in both soluble and membrane-bound forms by the malignant B cells of NHL samples [128]. Interestingly, these cells also have the unique ability to trap soluble TGFβ on their cell surface in order to protect it from degradation and keep it in its active form [128]. The full downstream inhibitory effects of these cytokines, including T cell exhaustion and induction of regulatory cells, have been reviewed elsewhere [129]. However, RNA fingerprinting analysis demonstrates that CD4+ T cells in HL samples are under the distinct transcriptional influence of TGFβ signaling, so it is clear that these cytokines do play an important role in the reprogramming of infiltrating immune effector cells [130].

Vascular endothelial growth factor (VEGF) is also highly expressed by malignant lymphoma cells, correlating with disease progression and a negative response to therapy [131,132,133]. Circulating serum levels of VEGF are also elevated in lymphoma patients and a predictor of poor prognosis [134,135,136]. Mouse models demonstrate that engraftment of NHL directly correlates with tumor VEGF production, and targeting the VEGF pathway with neutralizing antibodies results in inhibition of tumor growth [137,138]. The mechanisms of this tumorigenic activity are classically thought to be related to angiogenesis, vascular function, and direct effects on tumor cell survival, but there is growing evidence that VEGF is also important in establishing immune tolerance to cancers [139]. In the context of lymphoma, tumor-derived VEGF has been shown to impair DC maturation [140] as well as directly inhibit the proliferation and cytolytic activity of effector T cells [54].

### 4.4. Chemokines

Chemokines are chemoattractant cytokines that regulate the migration of specific populations of cells during homeostasis and when mounting an immune response; however, this tightly regulated system is often hijacked by lymphomas and associated cells within the microenvironment to attract immunosuppressive cells to the tumor. For example, CCL2/MCP-1 expression is induced in healthy donor mesenchymal stromal cells by co-culture with malignant FL cells, possibly via secretion of TNF by the tumor cells. This leads to the recruitment of monocyte precursors, which go on to develop a TAM-like phenotype [114]. The fact that NHL patients with higher expression of CCL2 have shorter survival times is consistent with this idea of increased resistance to immunity [141]. CXCL12, in addition to recruiting NHL cells to peripheral lymphoid organs [142,143], can likewise attract macrophages expressing CXCR4 to the hypoxic areas of tumors [144]. NHL biopsy specimens also expressed significantly high levels of CCL22, suggesting a possible mechanism for recruitment of immunosuppressive CCR4+ Tregs to the tumor [74]. Indeed, culture supernatants from these lymphoma cells induced chemotaxis and migration of Tregs that was significantly attenuated by a neutralizing anti-CCL22 antibody [74].

The reactive cellular infiltrate that characterizes Hodgkin’s lymphomas also seems to be at least partly due to the expression of chemokines by HRS cells. For example, CCL5/RANTES expression by HRS cells was found to recruit mast cells and eosinophils [145]. Not only do these mast cells send proliferation signals to the HRS cell via the CD30-CD30L interaction, but they also are a significant source of Th2 cytokines [146]. Interestingly, HRS cells also express the receptor CCR5, suggesting an autocrine growth signaling pathway [147]. High expression of the chemokine CCL17/TARC in HRS cells can also explain the predominantly Th2 infiltrate found in cHL [148]. Analysis of the secretome of HRS cells confirmed these findings and implicated many others, including fractalkine/CX3CL1, which can contribute to the recruitment of monocytes [149]. Taken together, HRS cells seem to use chemokines to actively recruit immune cells that both provide growth signals and skew the microenvironment away from an anti-tumor Th1 response.

### 4.5. Therapeutic Potential

Although the severe side effects of systemic cytokine therapies often limit their potential, several agents are under investigation for their specific ability to reverse immunosuppression and drive anti-tumor immunity in lymphomas. IL-21, in addition to its direct induction of apoptosis [150], has been shown to increase anti-tumor immune activity in preclinical models, partly by the suppression of Treg proliferation [151] and partly by the enhanced activity of ADCC effectors [152]. In a phase I trial of IL-21 and rituximab, 33% of patients with rituximab-resistant disease responded, suggesting distinct clinical benefit from IL-21 [153].

In contrast, treatment of B cell NHL with IL-12, a potent driver of cytotoxic T cells, NK cells, and Th1 cell-mediated immunity, in combination with rituximab did not result in a better response rate than treatment with rituximab alone [154]. It was later found that although IL-12 induces IFNγ production and counteracts Th2 skewing within the tumor microenvironment, long-term exposure can result in upregulation of Tim-3 and T cell exhaustion, thereby negating any clinical benefit [155].

The production of sIL-2Rα by lymphoma cells suggests that supplementation with IL-2 may be a valid treatment strategy; however, the dual role of IL-2 in expanding both cytotoxic effector cells as well as Tregs complicates its efficacy as a therapy on its own. Indeed, multiple trials report that treatment with IL-2 provides little additional clinical benefit to treatment with rituximab even though immunophenotyping demonstrates increased levels of circulating effector cells and ADCC activity [156,157,158]. However, IL-2 can play a crucial role in other successful treatment strategies such as adoptive T cell transfer [159].

In the context of lymphoma, plerixafor is being explored as a way to antagonize CXCR4 and disrupt a chemokine axis crucial to the homing of cancer cells to lymphoid tissues [160]. Preclinical studies suggest CXCR4 antagonists may enhance anti-tumor activity by blocking interactions with stromal cells mediating immune protection and tumor survival [161,162]. It is quite possible that inhibiting the CXCL12/CXCR4 pathway may also reduce the recruitment of immunosuppressive macrophages [144].

Similarly, VEGF has been primarily targeted in lymphomas for its role in angiogenesis, but it may also have immune-mediated mechanisms of promoting tumorigenesis. Unfortunately, multiple trials have concluded that the addition of bevacizumab, a VEGF-blocking antibody to standard therapies significantly increases toxicity while providing minimal clinical benefit [163,164,165,166,167,168]. The one exception may be patients with relapsed FL, who benefited from longer progression-free survival after treatment with both bevacizumab and rituximab [169].

## 5. Perspectives

**Figure 1:**
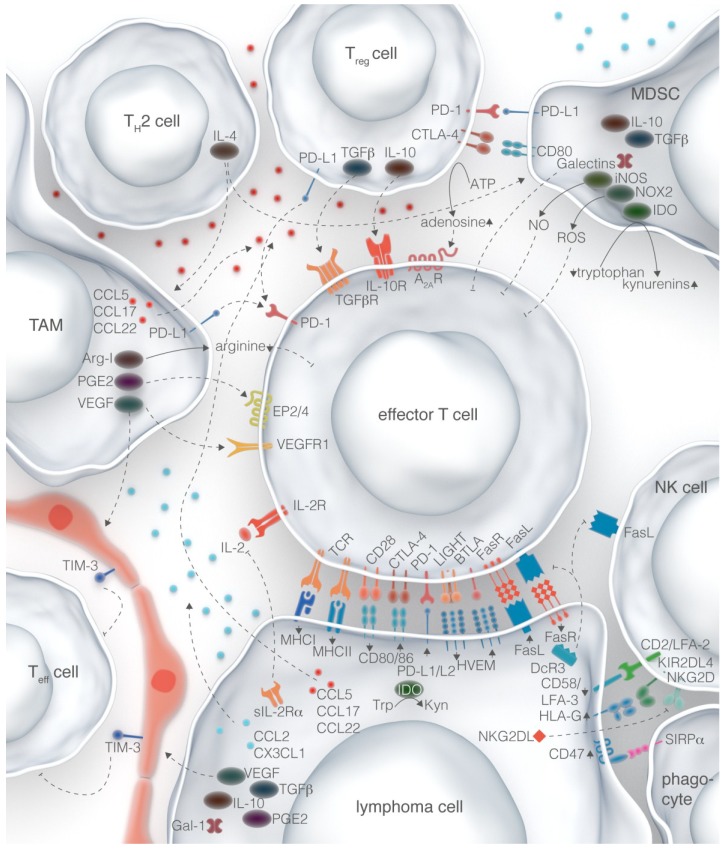
Immune evasion mechanisms in the lymphoma microenvironment. Lymphoma cells can suppress anti-tumor immune cells (e.g., effector T cells, NK cells, and phagocytes) directly through surface-mediated mechanisms as well as secreted factors. Chemokines expressed by the lymphoma cell can also recruit regulatory cells (e.g., tumor-associated macrophages, myeloid-derived suppressor cells, Tregs, and Th2-polarized cells) that form a complex network of interactions to maintain a tolerogenic microenvironment and allow the lymphoma cell to escape detection.

Incredible advances have been made over the past two decades regarding our understanding of interactions between the tumor and the immune system. The success of some therapies that have been translated to the clinic confirm that targeting these interactions is an effective approach to treating lymphomas; however, it has become readily apparent that there is no master regulator of immunosuppression, and lymphomas instead utilize many different mechanisms to reprogram the immune milieu both within the microenvironment and systemically (Figure 1). Therefore, the most successful treatment strategies for lymphomas may be to target multiple immune evasion pathways at the same time or to combine them with more conventional treatments like chemotherapy, radiation, stem cell transplantation, or other forms of immunotherapy.

Development of the next generation of immunomodulatory therapies will require a systems approach to studying the tumor microenvironment in order to deconvolute the complex network of interactions that occur not only between tumor cells and their immune counterparts, but also within immune cells in the context of the tumor. Dissecting the kinetics of such interactions will also be valuable. Recent advances in immunophenotyping technologies such as mass cytometry, in addition to declining costs for high-throughput sequencing, have made such studies more plausible than in the past. The wealth of new data that will come from these approaches will likely lead to an unprecedented leap in our understanding of lymphoma immune evasion and ultimately translate into the development of better treatments for our patients.
